# Epidemiology and molecular characterization of multidrug-resistant Gram-negative bacteria in Southeast Asia

**DOI:** 10.1186/s13756-016-0115-6

**Published:** 2016-05-04

**Authors:** Nuntra Suwantarat, Karen C. Carroll

**Affiliations:** Chulabhorn International College of Medicine, Thammasat University, Pathumthani, 12120 Thailand; Division of Medical Microbiology, Johns Hopkins University School of Medicine, Mayer B1-193, 600 North Wolfe Street, Baltimore, MD 21287-7093 USA; Microbiology Laboratory, Johns Hopkins Hospital, Baltimore, MD USA

**Keywords:** Gram-negative bacteria, Multidrug-resistance, Southeast Asia, Molecular, Epidemiology

## Abstract

**Background:**

Multidrug-resistant Gram-negative bacteria (MDRGN), including extended-spectrum β-lactamases (ESBLs) and multidrug-resistant glucose-nonfermenting Gram-negative bacilli (nonfermenters), have emerged and spread throughout Southeast Asia.

**Methods:**

We reviewed and summarized current critical knowledge on the epidemiology and molecular characterization of MDRGN in Southeast Asia by PubMed searches for publications prior to 10 March 2016 with the term related to “MDRGN definition” combined with specific Southeast Asian country names (Thailand, Singapore, Malaysia, Vietnam, Indonesia, Philippines, Laos, Cambodia, Myanmar, Brunei).

**Results:**

There were a total of 175 publications from the following countries: Thailand (77), Singapore (35), Malaysia (32), Vietnam (23), Indonesia (6), Philippines (1), Laos (1), and Brunei (1). We did not find any publications on MDRGN from Myanmar and Cambodia. We did not include publications related to *Shigella* spp., *Salmonella* spp., and Vibrio spp. and non-human related studies in our review. English language articles and abstracts were included for analysis. After the abstracts were reviewed, data on MDRGN in Southeast Asia from 54 publications were further reviewed and included in this study.

**Conclusions:**

MDRGNs are a major contributor of antimicrobial-resistant bacteria in Southeast Asia. The high prevalence of ESBLs has been a major problem since 2005 and is possibly related to the development of carbapenem resistant organisms in this region due to the overuse of carbapenem therapy. Carbapenem–resistant *Acinetobacter baumannii* is the most common pathogen associated with nosocomial infections in this region followed by carbapenem-resistant *Pseudomonas aeruginosa*. Although Southeast Asia is not an endemic area for carbapenem-resistant *Enterobacteriaceae* (CRE), recently, the rate of CRE detection has been increasing. Limited infection control measures, lack of antimicrobial control, such as the presence of active antimicrobial stewardship teams in the hospital, and outpatient antibiotic restrictions, and travel throughout this region have likely contributed to the increase in MDRGN prevalence.

## Background

The Southeast Asian region has a history of high prevalence of multidrug-resistant Gram-negative bacteria (MDRGN) including extended-spectrum *β*-lactamases (ESBLs) and multidrug-resistant (MDR) glucose-nonfermenting Gram-negative bacilli (nonfermenters), especially *Acinetobacter baumannii* and *Pseudomonas aeruginosa* [1, 2]. In addition, carbapenem-resistant organisms (CRO) have recently emerged and spread to Southeast Asia [3]. The epidemiology and molecular characteristics of MDRGN have been reported from Brunei, Indonesia, Laos, Malaysia, the Philippines, Singapore, Thailand, and Vietnam [1, 3–8]. Carbapenem–resistant *Acinetobacter baumannii* (CRAB) is the most common CRO associated with nosocomial infection in this region followed by carbapenem-resistant *Pseudomonas aeruginosa* (CRPA) [3–6]. Although Southeast Asia is not an endemic area of carbapenem-resistant *Enterobacteriaceae* (CRE), recently, the rate of CRE detection has been increasing [3, 4]. CREs are particularly concerning as these organisms are often disseminated by plasmids and have the potential to spread between patients causing outbreaks in several countries [1, 3–6]. We review and summarize current critical knowledge on the epidemiology and molecular characteristics of MDRGN organisms in Southeast Asia.

## Methods

### Literature search, definition and selection strategy

PubMed searches were performed for publications prior to 10 March 2016 with the term related to “MDRGN definition” combined with specific Southeast Asian country names (Thailand, Singapore, Malaysia, Vietnam, Indonesia, Philippines, Laos, Cambodia, Myanmar, Brunei). For epidemiologic purposes, we defined MDRGN as Gram-negative bacteria that are resistant to at least 3 classes of antimicrobial agents [1, 2]*.* We searched the terms Multidrug-resistant gram-negative bacteria, MDR Gram-negative bacteria, ESBL, KPC, NDM, VIM, IMP, MBL, CRE, *Acinetobacter, Pseudomonas.*

## Results and Discussion

There were a total of 175 publications from the following countries: Thailand (77), Singapore (35), Malaysia (32), Vietnam (23), Indonesia (6), Philippines (1), Laos (1), and Brunei (1). We did not find any publications on MDRGN from Myanmar and Cambodia. We did not include publications related to *Shigella* spp., *Salmonella* spp., and *Vibrio* spp. and non-human related studies in our review.

English language articles and abstracts were included for analysis. After the abstracts were reviewed, data on MDRGN in Southeast Asia from 54 publications were further reviewed and included in this study.

### Epidemiology and molecular characteristic of MDRGN in Southeast Asia

**Fig. 1:**
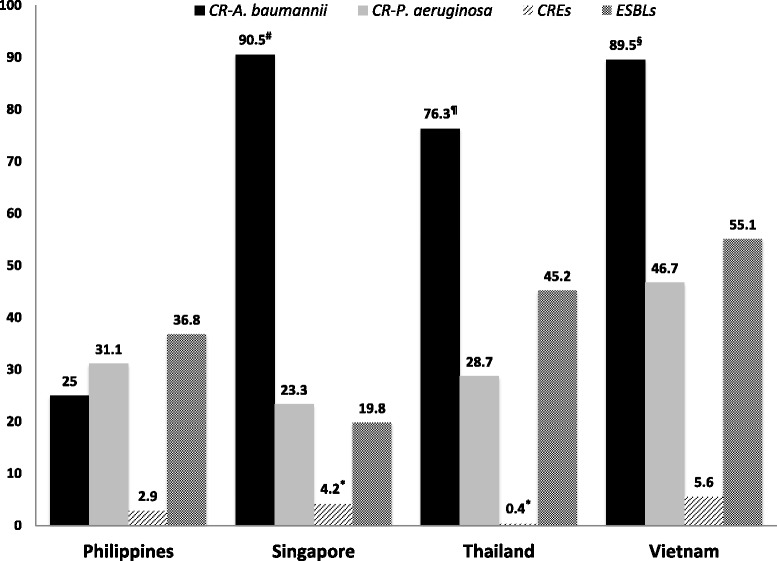
Prevalence (%) of extended-spectrum β-lactamases (ESBLs) and carbapenem-resistant organisms by country in Southeast Asia, adopted from reference 5 (COMPACT II study). The organisms were obtained during April – July 2010, from 5 Centers in Asia-Pacific countries including New Zealand (data not shown), the Philippines (3 centers, 16 *A. baumannii* isolates, 90 *P. aeruginosa* isolates, 70 *Enterobacteriaceae* isolates), Singapore (3 centers, 21 *A. baumannii* isolates, 120 *P. aeruginosa* isolates, 96 *Enterobacteriaceae* isolates), Thailand (10 centers, 59 *A. baumannii* isolates, 296 *P. aeruginosa* isolates, 239 *Enterobacteriaceae* isolates) and Vietnam (3 centers, 19 *A. baumannii* isolates, 90 *P. aeruginosa* isolates, 71 *Enterobacteriaceae* isolates). There are small numbers of *A. baumannii* isolations tested from reference 5. Prevalence of CRAB in other studies are ^#^70.5-91 % (Singapore) [6, 43, 44], ^¶^46.7-80 % (Thailand) [29–31] and ^§^more than 90 % (Vietnam) [48]. *Recent studies have been shown the increasing prevalence of CRE in Singapore and Thailand [1, 3, 4, 6, 22]. Abbreviation; CR, carbapenem–resistant; CRE, carbapenem-resistant *Enterobacteriaceae*; ESBLs, extended-spectrum *β*-lactamases

Overall, the prevalence of MDRGN bacteria varies by countries, institutions, and time of the studies. There were some publications on the prevalence of MDRGN bacteria in Southeast Asia but the studies’ limitations were related to small numbers of isolates tested in each country. The Comparative Activity of Carbapenem Testing (COMPACT) II study during April – July 2010 revealed a high prevalence of MDRGNs including ESBLs and CRO in Southeast Asia (Fig. 1) [5]. This study surveyed the carbapenem susceptibility against 1260 major Gram-negative organisms isolated from hospitalized patients at 20 centers in 5 Asia-Pacific countries (New Zealand, the Philippines, Singapore, Thailand and Vietnam). Amongst *Enterobacteriaceae* isolates, 39.4 % of 436 isolates tested (*Escherichia coli, Klebsiella pneumoniae, Klebsiella oxytoca* and *Proteus mirabilis*) were positive for ESBL production, with the highest rate in Vietnam (55.1 %) followed by Thailand (45.2 %). There were no ESBLs found in clinical isolates from New Zealand. ESBL-producers were more commonly isolated from intensive care unit (ICU) patients than non-ICU patients in the Philippines (58.8 % vs. 27.5 %) and Vietnam (81.0 % vs. 43.8 %). Among CRO isolates, the CRAB detection rate among clinical isolates was 73 % followed by a CRPA detection rate of 29.8 %. In contrast, the rate of CRE detection was only 2.8 % [5]. In a separate report, Menders et al. [1] reported the results of the Regional Resistance Surveillance program susceptibility rates from 12 Asia-Pacific countries (APAC) in 2011. Most of this surveillance data came from Indonesia, the Philippines, and Thailand and a small sampling of data came from Malaysia and Singapore. Among 310 strains, 96 isolates from Indonesia, the Philippines, and Thailand expressed the ESBL-resistance phenotype; the ESBL production rate in *E. coli* was 59.4 % (APAC regional rate, 48.0 %) and the highest rate occurred among Indonesian isolates (71.0 %). Also, the prevalence of ESBL-production in *Klebsiella* was 46.7 % (APAC rate, 47.0 %) and the highest rate occurred among Indonesian isolates (64.0 %). The CTX-M-series enzymes have become the dominant ESBL-type in this region [1, 9, 10].

The OXA-type carbapenemase gene, *bla*_OXA-23_ is a predominant *β*-lactamase gene among *A. baumannii* isolates in this region and belongs to global clone 2 [11, 12]. Two global clones of *A. baumannii* have been reported (global clone 1 and global clone 2) worldwide [10]. Global clone 2 corresponds to clonal complex 92 (CC92) in the multilocus sequence typing (MLST) scheme of Bartual et al. and Woodford et al. [11, 12]. Global clone 2 has emerged in Europe and spread throughout *A. baumannii* isolates in Asian countries, including South Korea and China and Australia [11]. In addition, the *bla*_OXA-40_, and *bla*_OXA-58_ have been reported in a few *A. baumannii* isolates in this region [3, 6, 11, 12]. In *P. aeruginosa*, carbapenem resistance is multifactorial and involves non-carbapenamase mechanisms such as porin changes (OprD gene mutation) and a combination of efflux pump and AmpC *β*-lactamase hyperproduction. Metallo *β*-lactamase-production (MBL) has been reported in CRPA and includes IMP-type metallo-*β*-lactamase (*bla*_IMP_) and Verona integron-mediated MBL (*bla*_VIM_) [3, 6, 11–15]. Among CRE isolates, New Delhi MBLs (NDM) have emerged and predominate in several countries in this region [6, 16–20]. Other MBL genes such as *bla*_IMP,_*bla*_VIM_ and *bla*_OXA_ have been reported in some countries [6, 19, 21–23]. *Klebsiella pneumoniae* carbapenamase (KPC)-producing *Enterobacteriaceae* is less common in Southeast Asia and officially reported only from Singapore and Thailand [22, 24]. This finding is in contrast to data from North America and Europe where *bla*_KPC_ is the most common *β*-lactamase gene reported among CRE isolates [6, 22, 24]. In addition, non-carbapenamase mechanism such as outer member protein changes (Omp gene mutation) and AmpC *β*-lactamase hyperproduction are common mechanisms of resistance in CRE isolates [3, 6, 25].

### Thailand

The majority of epidemiology and molecular studies of MDRGN in Thailand were performed on isolates from hospitalized patients [1, 5, 10, 26]. Recently, a cross sectional study from an academic tertiary care hospital in Thailand between February and May 2012 revealed a high prevalence of MDRGN (48.8 %). The percentage of MDRGN was 37.8 % for ESBL-producers, 39.3 % for CRPA, and 88.7 % for CRAB [10]. Infections caused by MDRGN were associated with admission to medical wards, were of respiratory tract origin and hospital onset of infection. Using multivariate analysis, the only significant risk factor of MDRGN infection was previous antibiotic use within 1 year (adjusted odds ratio 6.818, 95 % CI = 1.337–34.770) [10]. ESBLs of the *bla*_CTX-M type_ are highly endemic in Thailand, especially among hospital-associated isolates [10, 26]. Kiratisin et al. [10], performed a molecular study on 362 isolates of ESBL-producing *E. coli* (n = 235) and ESBL-producing *K. pneumoniae* (n = 127) collected from patients with hospital-associated infection at two major university hospitals in Thailand from December 2004 to May 2005. A total of 87.3 % of isolates carried several *bla* genes. The prevalence of *bla*_CTX-M_ was 99.6 % for ESBL-producing *E. coli* (CTX-M-14, -15, -27, -40, and -55) and 99.2 % for ESBL-producing *K. pneumoniae* (CTX-M-3, -14, -15, -27, and -55). Up to 77.0 and 71.7 % of ESBL-producing *E. coli* and ESBL-producing *K. pneumoniae*, respectively, carried *bla*_TEM-1_. ESBL-producing *K. pneumoniae* carried *bla*_SHV_ at 87.4 % (SHV-1, -2a, -11, -12, -27, -71, and -75) but only at 3.8 % for ESBL-producing *E. coli* (SHV-11 and -12). The *bla*_VEB-1_ and *bla*_OXA-10_ were also found in both ESBL-producing *E. coli* (8.5 and 8.1 %, respectively) and ESBL-producing *K. pneumoniae* (10.2 and 11.8 %, respectively). None of the isolates were positive for *bla*_PER_ and *bla*_GES_. Pulsed-field gel electrophoresis (PFGE) analysis demonstrated that there was no major clonal relationship among these ESBL isolates.

There are limited data on clinical and molecular epidemiology of community-onset (CO) ESBLs in Thailand. Apisarnthanarak et al. [27], performed a case–control study to evaluate risk factors for CO-ESBL-producing *E coli* infections (n = 46). Controls (n = 138) were patients without infections. Patients with prior ESBL colonization and recent antibiotic exposures, especially to third-generation cephalosporins and fluoroquinolones, were at risk for CO-ESBL-producing *E coli* infection. The plasmid carrying the *bla*_CTX-M-15_ gene was identified in 52 %. In addition, evidence of a high prevalence of ESBL-producing *E. coli* isolates recovered from healthy individuals and foods along the food production chain from farms to consumers, and in the environment has been documented in selected areas in Thailand. Among 544 healthy adult food factory workers, 906 bacterial isolates were recovered from rectal swab screening cultures and 75.5 % were positive for ESBL-producing *E. coli*. Moreover, 77.3 % of *E. coli* isolates collected from 30 healthy animal farm workers were ESBLs [28].

*A. baumannii* infections represent a key healthcare issue in Thailand. Data from the National Antimicrobial Resistance Surveillance Thailand (NARST) detected a dramatic increase in CRAB from 2.1 % in 2000 to 46.7 % in 2005 [29]. Prevalence rates of colonization and infections of CRAB were reported to be up to 80 % in several hospitals in Thailand [30, 31]. Infection-related mortality could be as high as 63.0 % in the patients with *A. baumannii* bacteremia [31]. In addition, several studies from tertiary care and academic hospitals in Thailand have reported a high rate and clonal infection from CRAB throughout the country [10, 32–35]. CRAB occurs mainly as a result of the *bla*_OXA_ gene, and MBL gene acquisition [12, 36]. The *bla*_OXA-23_ is a major resistance determinant among CRAB isolates in Thailand and has been reported related to global clone 2 [12, 36–38].

The prevalence of MDR-*P. aeruginosa* clinical isolates was constant among 28 hospitals participating in the NARST program in Thailand from 2000 to 2005. The most common sites of isolation included sputum, pus, and urine. The prevalence of MDR-*P. aeruginosa* ranged from 20 % to 30 % of the isolates [39]. Khuntayaporn et al. [13] performed susceptibility tests on 261 clinical isolates of MDR-*P. aeruginosa* (collected during 2007-2009) from eight tertiary hospitals across Thailand. Approximately 71.7 % were found to be MDR-*P. aeruginosa*. The results showed that the meropenem resistance rate was the highest reaching over 50 % in every hospital. Additionally, the type of hospital was a major factor affecting the resistance rate, as demonstrated by significantly higher rates of CRO among university than regional hospitals. CRPA clinical isolates in Thailand possess multifactorial resistance mechanisms [14, 40]. The decreased expression of OprD mRNA was the most common mechanism (93.7 %). This mechanism was associated with the presence of OprD mutations causing frameshift or translational stop and the reduction of antibiotic transportation in to the CRPA cell. MBL production was identified in 24 isolates (18.5 %) and weakly positive in 12 isolates (9.2 %) including *bla*_IMP-1_, *bla*_IMP-14_ and *bla*_VIM-2_. AmpC *β*-lactamase hyperproduction had the lowest prevalence rate (4 %) [14]. This study indicates that the loss of OprD porin protein was the most common mechanism for imipenem resistance in *P. aeruginosa* clinical isolates (98 %) [14] which is consistent with another study [40].

There are few data on CRE prevalence in Thailand. However, Rimrang et al. [16], reported the emergence of NDM-1- and IMP-14a-producing *Enterobacteriaceae* in Thailand. A total of 4818 *Enterobacteriaceae* clinical isolates, collected between October 2010 and August 2011, were screened for the presence of carbapenemases. The study revealed 2 other isolates each of *Escherichia coli, Klebsiella pneumoniae* and *Citrobacter freundii* carried *bla*_NDM-1_ and 2 other isolates of *K. pneumoniae* carried *bla*_IMP-14a_. The DNA fingerprints revealed that all isolates were different strains except for clonal strains of *C. freundii*. All MBL producers were susceptible to colistin and tigecycline. Interestingly, 6 NDM-producing isolates were recovered from the urine of 3 patients, who had no history of travel outside Thailand. Netikul et al. [24], reported a novel KPC-13*-*producing CRE in Thailand. In addition, Kiratisin et al. [41], investigated the genetic characteristics of plasmid-mediated *β*-lactamase among non-*Escherichia*, non-*Klebsiella Enterobacteriaceae* that were non-susceptible to at least a broad-spectrum cephalosporin. From 598 isolates, 143 isolates (23.9 %) were resistant to a broad-spectrum cephalosporin, amongst which 142 (99.3 %) and 99 (69.2 %) isolates carried ESBL and AmpC *β*-lactamase genes, respectively. The *bla*_KPC_ was not detected in isolates with reduced susceptibility to carbapenems.

### Singapore

The largest gram-negative resistance problem in Singapore hospitals is ESBL-producing *Enterobacteriaceae* particularly *Klebsiella* spp. and *E. coli* [1, 5, 6]. ESBL-producing *Enterobacteriaceae* were first reported in Singapore in the late 1990s and increased rapidly up to 35 % - 40 % over time. Similar to observed trends in other countries, TEM and SHV type ESBLs have spread to Singapore. These are being replaced by the newer CTX-M type ESBLs [1, 6]. CTX-M type ESBLs are currently a major resistance contributor, especially in nosocomial infections. *K. pneumoniae* isolates were found to be carrying genes for CTX-M-9 type and CTX-M-1 type ESBLs, and *E. coli* possess a CTX-M-2 type ESBL. Recently, community associated infections have also been reported and associated with CTX-M type ESBLs [6]. Finally, some CTX-M ESBLs may also be associated with carbapenem resistance in combination with porin loss or efflux [6, 42]. Nevertheless, a recent study concluded that the worldwide spread of the gene for the *bla*_CTX-M-15_ is due to epidemic *E. coli* clones belonging to Achtman’s MLST 131 and ST405 [43].

In Singapore, carbapenem resistance is more common in *Acinetobacter* spp. and *P. aeruginosa* than in *Enterobacteriaceae*. However, there are several reports of new CRE genes that have recently been described in clinical isolates [6, 17, 44]. The discovery of these new genes is likely related to updated national surveillance data on CRO and more molecular characterization studies performed in Singapore compared to other countries in Southeast Asia. In addition, Singapore is a center of communication and commercial trading and travelling in this region. These factors might be also contributed to the spreading of CROs from travellers [44].

CRAB have emerged as important pathogens in Singapore since 1990. The prevalence of CRAB has been increasing over the time [6]. Tan et al. [44] found 98 isolates of CRAB (70.5 %) from a total of 171 *Acinetobacter* spp. isolates (139 *A. baumannii* isolates) collected from 6 hospitals in Singapore during 2006-2007. The rate of carbapenem resistance in *A. baumannii* (70.5 %) was higher than in other *Acinetobacter* spp. (25.0 %) [44]. The *bla*_OXA-23_ genes were detected in most of the CRAB isolated (91 %) in a Singapore hospital, while *bla*_IMP-4_ and *bla*_OXA-58_ genes were also detected in a few isolates [6, 45].

CRPA has emerged in Singapore during the same time as CRAB. Tan et al. [45] reported that 11.2 % of 188 isolates of *P. aeruginosa* collected during 2006–2007 were resistant to meropenem. Nevertheless, like the situation in other countries, the resistance in CRPA is related to multifactorial mechanisms. Acquired MBL genes represented 1.7 % of all *P. aeruginosa* isolates collected at Singapore General Hospital during 2001. The common MBLs in CRPA are *bla*_IMP-1_, *bla*_IMP-7_, and *bla*_VIM-6_ which also have been previously reported in Japan, Canada, and Malaysia [6].

Although mechanisms of resistance in CRE seems to be largely due to non-carbapenamase mechanisms, the emergence of CRE isolates that carry transferable carbapenamase genes have been reported from hospital and community settings in Singapore. MBLs, especially NDM-1, are a major mechanism of resistance, [6, 17, 22, 44, 46, 47]. Koh et al. [22], reported isolates of *K. pneumoniae, E. coli, Enterobacter cloacae* and *Citrobacter* spp. carried a variety of carbapenemase genes including *bla*_IMP-1_, *bla*_IMP-4_, *bla*_NDM-1_, *bla*_NDM-7_, *bla*_OXA-48_, *bla*_OXA-181_ and *bla*_KPC-2_. Apart from *K. pneumoniae* with *bla*_OXA-181_, and some *K. pneumoniae* with *bla*_NDM-1_, the other isolates were not clonal using PFGE analysis. Teo et al. [17] molecularly characterized 12 NDM-1 producing clinical *Enterobacteriaceae* (*K. pneumoniae, E. coli, E. cloacae*) isolates from 4 general hospitals in Singapore. Interestingly, none of the patients had a travel history to countries where NDM-1 has been reported. None of the isolates in the Teo study were clonally related using PFGE analysis [17]. *Enterobacteriaceae* carrying *bla*_KPC_ are not common in Singapore. The first KPC-producing *K. pneumoniae* isolate was reported from a study in 2011. This isolate carried *bla*_KPC-2_ and was identified as Pasteur’s MLST ST 11 [47].

### Vietnam

The Study for Monitoring Antimicrobial Resistance Trends (SMART 2009–2011) about antimicrobial susceptibility and ESBL rates in aerobic gram-negative bacteria causing intra-abdominal infections in Vietnam, reported high ertapenem MIC90 values for *A. baumannii,* and *P. aeruginosa* (>4 μg/mL) [48]. In addition, among the species collected, *E. coli* (48.1 % ESBL-positive) and *K. pneumoniae* (39.5 % ESBL-positive) represented the majority (46.4 %) of the isolates submitted for this study. Ertapenem MIC90 values were lowest for these 2 species at 0.12 and 0.25 μg/mL and remained unchanged for ESBL-positive isolates. Imipenem MIC90 values were also the same for all isolates and ESBL-positive strains at 0.25 and 0.5 μg/mL, respectively [48]. Van et al. [49] performed antimicrobial susceptibility testing and molecular characterization on 66 *A. baumannii* complex clinical isolates recovered during 2009 at the National Hospital of Tropical Diseases (NHTD), a referral hospital in Hanoi, Vietnam. Most isolates were collected came from lower respiratory tract specimens from ICU patients. More than 90 % of the isolates were CRAB. Moreover, 25.4 % were resistant to all tested *β*-lactams, quinolones and aminoglycosides. All isolates remained susceptible to colistin. Unlike CRAB, there is limited data on CRPA prevalence in Vietnam. A novel *bla*_IMP-51_ has been reported [50]. In Vietnam, NDM-1-producing *E. coli* and *K. pneumoniae* have emerged since 2010. Both organisms were recovered from two patients admitted to a surgical hospital. These patients had no history of travel outside Vietnam [20]. In addition, Trung et al. [19] reported that *A. baumannii* clinical isolates carried the ESBL gene (PER-1) and genes from the *bla*_OXA_ families (OXA-23, OXA-24 and OXA-58). Interestingly, one *A. baumannii* that carried *bla*_NDM-1_ was recovered from a suspected surgical wound infection using a novel in-house multiplex PCR assay.

### Malaysia

In 2009, Lim et al. [51], performed a molecular characterization on 47 *E. coli* isolates from various public hospitals in Malaysia. All isolates were susceptible to imipenem whereas 36 (76.6 %) were MDR *E. coli* (resistant to 2 or more classes of antibiotics). The majority of ESBL-producing *E. coli* (87.5 %) harbored the *bla*_TEM_ gene. Other ESBL-encoding genes detected were *bla*_OXA_, *bla*_SHV_, and *bla*_CTX-M_. Integron-encoded integrases were detected in 55.3 % of isolates. In addition, another study from the same leading author performed molecular characterization on 51 strains of *K. pneumoniae* isolated from the same hospitals in Malaysia. The majority of the strains (98 %) were susceptible to imipenem whereas 27 (52.9 %) were MDR *K. pneumoniae*. Forty-six of the *K. pneumoniae* strains harbored *bla*_SHV_, 19 harbored *bla*_CTX-M_, 5 harbored *bla*_OXA-1_ and 4 harbored *bla*_TEM-1_ [52].

Among a total of 54 *A. baumannii* isolates from the main tertiary hospital in Terengganu, Malaysia, 39 (72.2 %) were CRAB, whereas 14 (25.9 %) were categorized as extensively drug resistant (XDR) with additional resistance to polymyxin B [53]. CRPA prevalence in Malaysia was reported as 21 % [54]. Khosravi et al. [15] performed molecular characterization on 90 isolates of imipenem-resistant *P. aeruginosa* clinical isolates collected during 2005 to 2008 from the University of Malaysia Medical Center. A multiplex PCR assay detected 32 isolates with positive MBL genes including; *bla*_IMP-7_ (12 isolates), *bla*_IMP-4_ (2 isolates), *bla*_VIM-2_ (17 isolates), and *bla*_VIM-11_ (1 isolate). For CRE prevalence in Malaysia, a total of 321 *K. pneumoniae* isolates collected during April 2010-June 2012 from academic hospitals were characterized. Thirteen isolates (4.0 %) were CRKP and the majority of them were resistant to all tested antibiotics except colistin and tigecycline. Among seven different carbapenemase genes studied (*bla*_KPC_, *bla*_IMP_, *bla*_SME_, *bla*_NDM_, *bla*_IMI_, *bla*_VIM_, and *bla*_OXA_), only *bla*_IMP4_ (1.87 %) and *bla*_NDM1_ (2.18 %) were detected in this study [18]. In addition, another study also confirmed imipenem-resistance in *K. pneumoniae* in Malaysia due to loss of OmpK36 (outer membrane protein) coupled with AmpC *β*-lactamase hyperproduction [25].

### Other countries

There are limited data on prevalence and epidemiology of MDRGN in other countries in Southeast Asia. Indonesia has rates of ESBLs greater than the overall APAC average and for most nations in Western Europe and the United States [1, 5]. In 2011, another study confirmed a high prevalence of CRO in ICU-Cipto Mangunkusumo Hospital in Indonesia. The prevalence of CRE, CRPA and CRAB are 27.6, 21.9, and 50.5 %, respectively. CRPA harboring the *bla*_IMP-1_ gene (5 %) were isolated from sputum specimens. Moreover, *bla*_NDM-1_ was detected in one *K. pneumoniae* isolated from sputum [23]. In the Philippines, an *E. coli* isolate carrying *bla*_IMP-26_ has been reported [21]. Antibiotic resistance has been little studied in Laos, where some antibiotics are available without restriction, but others such as carbapenems are not available. Stoesser et al. [7] reported 92 children (23 %) were colonized with ESBL-producing *E. coli* carrying *bla*_CTX-M_ and *K. pneumoniae* carrying *bla*_SHV_ or *bla*_CTX-M_, which were frequently resistant to multiple antibiotic classes. Using multivariate random-effects model, ESBL colonization was associated with prior antibiotic use within 3 months. Additional whole genome sequencing studies suggested the transmission of ESBLs in both childcare facilities and community settings. Tojo et al. [8], reported a case of CRAB isolates obtained from a returned traveler from Brunei. This isolate was a second case of *A. baumannii* harboring *bla*_OxA-23_ reported from Japan. There was no publication on MDRGN reported from Myanmar and Cambodia.

This review has some limitations. Epidemiology and prevalence of MDRGN bacteria is a dynamic issue worldwide and especially in Southeast Asia. However, a systematic review could not be performed due to limitations of available data and the difficulty in standardizing all data. The lack of international collaboration on antimicrobial surveillance studies might have an effect on the accuracy of the actual prevalence of MDRGN bacteria in this region. Molecular studies on MDRGN bacteria are not routinely performed in microbiology laboratories in several countries due to limited resources. Lastly, the formal languages in this region are diverse but we only reviewed available English literature as is commonly used for scientific publications.

## Conclusions

In summary, MDRGNs are a major contributor of antimicrobial-resistant bacteria in Southeast Asia. The high prevalence of ESBLs has been a major problem since 2005 and is possibly related to the development of carbapenem resistant organisms in this region due to the overuse of carbapenem therapy to treat those infections. Prevalence of CROs in this region, including CRAB, CRPA and CREs, is rising. The high prevalence of MDRGNs in the hospital and community have precipitated development of CROs. Limited infection control measures, lack of antimicrobial control, such as the presence of active antimicrobial stewardship teams in the hospital, and outpatient antibiotic restrictions, and travel throughout this region have likely contributed to the increase in MDRGN prevalence. Thus, improving infection control practices and laboratory detection, along with judicious use of antimicrobial agents, and national surveillance could impact MDRGNs spread in this region.
